# Efficacy and safety of glucagon-like peptide 1 agonists for Parkinson's disease: a systematic review and meta-analysis

**DOI:** 10.1055/s-0045-1806824

**Published:** 2025-04-27

**Authors:** Luis O. S. Nogueira, Roberto A. S. V. Mazetto, Maria L. R. Defante, Vânio L. J. Antunes, Ocílio Ribeiro Gonçalves, Angela Maria Sandini Corso, Marcus V. Della Coletta, Dayany Leonel Boone, Walderico Silva Machado Filho, Vanderci Borges, Henrique Ballalai Ferraz

**Affiliations:** 1 Universidade do Estado do Amazonas, Escola Superior de Ciências da Saúde, Departamento de Medicina, Manaus AM, Brazil.; 2 Centro Universitário Redentor, Departamento de Medicina, Itaperuna RJ, Brazil.; 3 Universidade Federal de Ciências da Saúde de Porto Alegre, Porto Alegre RS, Brazil.; 4 Universidade Federal do Piauí, Centro de de Ciências da Saúde, Departamento de Medicina Especializada, Teresina PI, Brazil.; 5 Universidade Federal do Paraná, Setor de Ciências da Saúde, Curso de Medicina, Curitiba PR, Brazil.; 6 Universidade Federal de São Paulo, São Paulo SP, Brazil.; 7 Universidade Estadual de Campinas, Hospital de Clínicas, Campinas SP, Brazil.

**Keywords:** Glucagon-Like peptide 1, Parkinson Disease, Randomized Controlled Trial, Movement Disorders, Meta-Analysis

## Abstract

**Background** Recent research on Parkinson's disease (PD) therapy has highlighted glucagon-like peptide 1 (GLP-1) agonists as potential therapeutic agents. However, recent randomized controlled trials (RCTs) have shown mixed results regarding the use of this medication.

**Objective** To perform a meta-analysis comparing GLP-1 agonists with placebo or standard PD treatment in adult PD patients.

**Methods** We systematically searched the PubMed, Embase and Cochrane Central databases. The efficacy outcomes were assessed through the Movement Disorder Society Unified Parkinson Disease Rating Scale (MDS-UPDRS) and the 39-item Parkinson's Disease Questionnaire (PDQ-39). We also assessed adverse events. Dichotomous data were compared using the risk ratio (RR), and continuous endpoints were pooled using the mean difference (MD).

**Results** We included 4 RCTs, with a total of 514 patients. In every study, the Hoehn and Yahr stage was < 3. The pooled analysis demonstrated that the use of GLP-1 agonists was not associated with an improvement in the scores on parts I, II, III, and IV of the MDS-UPDRS at 6 and 12 months of follow-up. Neither did quality of life (PDQ-39) show significant differences among the groups, and a higher risk of gastrointestinal adverse events and weight loss was observed with the use of GLP-1 agonists. A subgroup analysis further confirmed the lack of clinical benefits of the intervention regarding all of these efficacy outcomes, and the intervention also significantly reduced result heterogeneity.

**Conclusion** In 1 year, GLP-1 agonists failed to improve motor and non-motor features of PD. Additional high-quality studies are needed to draw more robust conclusions about this treatment.

## INTRODUCTION

Parkinson's disease (PD) is a progressive neurological disorder whose pathophysiology involves dopaminergic depletion and accumulating effects of alpha-synuclein species. 1 The therapeutic approaches are aimed at dopamine replacement, and they focus on restoring dopaminergic activity to control motor symptoms. 2 Although these treatments can initially relieve symptoms, complex fluctuations and dyskinesias can develop over time, affecting the quality of life (QoL) and mobility of the patients. The glucagon-like peptide 1 (GLP-1) receptor is present in the brain's neuronal circuits, and agonist activity is thought to be anti-inflammatory by reducing microglial activation. 3 The current literature 4 suggests that these GLP-1 receptor agonists can influence several neuronal pathways, such as those responsible for neuroinflammation and mitochondrial function.

In this context, preclinical studies with PD models have demonstrated that GLP-1 agonist treatment can improve motor and nonmotor symptoms. Despite these effects, the real benefit of these drugs in PD is not well known. 4 There is evidence that people with type-2 diabetes have an increased risk of developing PD, although the precise role of antidiabetic medications, such as GLP-1 agonists, on PD risk lacks further elucidation. 5 To date, the efficacy and safety of GLP-1 analogs for PD treatment remain unanswered questions.

In a meta-analysis, Wang et al. 6 evaluated the use of antidiabetic drugs in the treatment of PD. The authors of this study, which included exenatide and pioglitazone as interventions, concluded that the former can reduce cognitive, motor, and nonmotor PD symptoms. However, only 2 trials involving 104 patients were available at that time. Recently, other large randomized controlled trials (RCTs) have been published, 7 8 which have included GLP-1 agonists as interventions for the treatment of PD, greatly increasing the population of randomized patients. These studies 7 8 have reported mixed and inconclusive results, raising questions about the overall efficacy of GLP-1 agonists. Therefore, we aimed to conduct a comprehensive, updated systematic review and meta-analysis of RCTs evaluating the efficacy and safety of GLP-1 receptor agonists in PD treatment.

## METHODS

The current meta-analysis was registered on the International Prospective Register of Systematic Reviews (PROSPERO) on April 19, 2024, under protocol ID CRD42024533414. 9 The study was performed in accordance with the Preferred Reporting Items for Systematic Reviews and Meta-Analysis (PRISMA) statement 10 and the recommendations of the Cochrane Collaboration Handbook for Systematic Reviews of Interventions. 11

### Eligibility criteria

To keep the sample as homogeneous as possible, the inclusion criteria were as follows:

- Adult patients diagnosed with PD, regardless of the disease stage;
- Inclusion of RCTs;
- Comparison between the use of a GLP-1 receptor agonist and placebo or standard PD treatment only; and
- Assessment of any of the endpoints of interest.

We excluded studies that were nonrandomized or that had overlapping patient populations.

### Search strategy

We performed an electronic datavase search on PubMed, Embase, and the Cochrane Central Register of Controlled Trials from inception to April 2024. We used the following search strategy: ( *parkinson* OR *parkinson's disease* ) AND ( *exenatide* OR *NLY01* OR *Glucagon-Like Peptide* OR *lixisenatide* OR *GLP-1* OR *semaglutide* OR *teduglutide* OR *liraglutide* OR *dulaglutide* ). Two investigators (LOSN and RASVM) independently performed the literature search. We excluded duplicates after the preselection process, in which we reviewed the abstract of each article to determine whether the study fit our research interest. All publications that were not excluded after preselection were read in full to further verify their eligibility. The investigators then compared the results and discussed and made corrections as necessary.

### Endpoints and data extraction

The endpoints included the score on part III (motor examination) of the Movement Disorder Society Unified Parkinson Disease Rating Scale (MDS-UPDRS), which was assessed in the on-medication state (that is, with the patient receiving a stable dose of dopaminergic medication to treat symptoms) and off-medication state (defined as an overnight washout of dopaminergic medication to treat symptoms), as well as the scores on part I (non-motor experiences of daily living), part II (motor experiences of daily living) and part IV (motor complications) of the same scale. 12 Lower numerical scores indicate lower levels of impairment in performance or function, while higher scores indicate higher levels of impairment. The 39-item Parkinson's Disease Questionnaire (PDQ-39), a self-report questionnaire, assesses how often patients with PD experience difficulties in 8 aspects of daily life; 13 it was used to assess QoL. Nausea, vomiting, diarrhea, constipation, and weight loss were the outcomes of interest regarding safety. Two authors (RASVM and VLJA) extracted outcome data independently, and disagreements were resolved by consensus.

### Quality assessment

The risk of bias in each study was assessed independently by two authors (MLRD and ORG) using the Cochrane tool to assess the risk of bias in randomized trials (RoB 2). 14 Disagreements were resolved through arbitration by a third author (LOSN). The risk of bias plot was created with the Risk of Bias Visualization (ROBVIS) tool ( **Supplementary Material Figure S6** –available at https://www.arquivosdeneuropsiquiatria.org/wp-content/uploads/2025/01/ANP-2024.0313-Supplementary-Material.docx ; online only). 15 A funnel plot to assess publication bias was not generated due to the small number of included trials (less than 10), in accordance with the Cochrane guidelines. 11

### Meta-analysis

Meta-analyses of the efficacy outcomes were performed by stratifying the trials into 2 groups based on their follow-up period: 6 months (including trials reporting outcomes at 24–26 weeks) and 12 months (including trials reporting outcomes at 48–52 weeks). Although active treatment ended between 36 and 52 weeks in all studies, an additional meta-analysis was conducted over a 14-month follow-up period (60 weeks) for part III of the MDS-UPDRS in the off-medication state to examine possible persistent effects of GLP-1 agonists beyond the duration of exposure.

All patients were analyzed according to the intention-to-treat principle. Different doses of the same drug were grouped together using the Cochrane's combining method. 11 Dichotomous data were compared using the risk ratio (RR) and continuous endpoints were pooled using the mean difference (MD) with 95% confidence intervals (95%CIs). Values of *p* < 0.05 were considered statistically significant. The Cochran's Q test and I ^2^ statistics were used to assess heterogeneity. Values of *p* < 0.10 and of I ^2^ > 25% were considered significant for heterogeneity. Finally, a subgroup analysis of placebo-controlled trials only was performed to investigate the source of heterogeneity. An investigation of the influence of age (younger or older than 60 years) on GLP-1 efficacy was intended, but it was not possible due to lack of comparable data. Restricted maximum likelihood (REML) random effects models were used for all outcomes, and the R (R Foundation for Statistical Computing, Vienna, Austria) software, version 4.3.2, was used for the statistical analyses.

## RESULTS

### Study selection and characteristics

**Figure 1:**
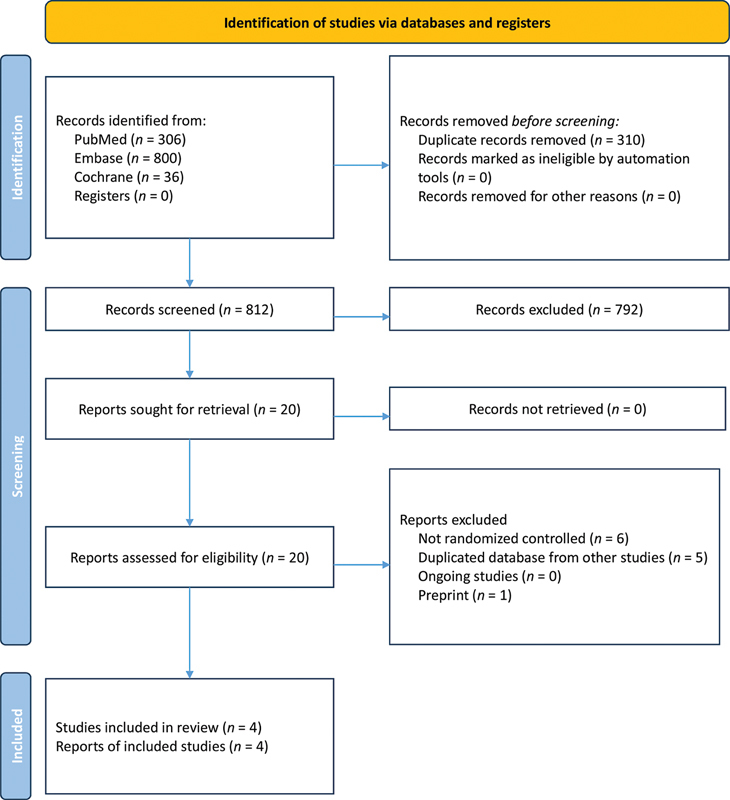
Preferred Reporting Items for Systematic Reviews and Meta-Analysis (PRISMA) flow diagram of study screening and selection.

We identified 1,142 reports in the initial database search ( Figure 1 ). Of these, 20 were fully screened according to the inclusion criteria, and 4 RCTs, 7 8 16 17 comprising 514 patients, were included in the final analysis. These studies, which were conducted between 2013 and 2024, used different GLP-1 agonists: lixisenatide, exenatide, and NLY01, a longer-lasting version of the latter. The mean age of the samples ranged from 57.8 to 62.1 years. The proportion of male participants in the intervention or control groups ranged from 56 to 83%. The Hoehn and Yahr stage (range: 1–5, with higher scores indicating worse disability) was lower than 3 in all studies. Other characteristics of the included studies are shown in Table 1 .

**Table 1:** Baseline characteristics of the included studies

Author (year)	Population	Medication	No. of patients	Male patients: n (%)	Mean age	Off-medication state- definition	Mean score on part III of the MDS-UPDRS off-medication	Hoehn-Yarh Stage: 1–2/2.5	Analysis
Athauda et al. 17 (2017)	Moderate PD	Exenatide And placebo	31; 29	22 (71); 22;(76)	61.6 ± 8.2; 57.8 ± 8.0	At least 8 hours before evaluation	32.8 ± 9.7; 27.1 ± 10.3	29/2; 29/0	6-, 12-, and 14 month follow-up
Aviles-Olmos et al. 16 (2013)	Moderate PD	Exenatide Cand conventional PD medication	20; 24	15 (75); 20 (83)	61.4 ± 6.0; 59.4 ± 8.4	Overnight period before evaluation	31.0 ± 11.2; 34.0 ± 16.1	14/6; 16/8	6-, 12-, and 14-month follow-up
McGarry et al. 7 (2024)	Early untreated PD	NLY01 (5 mg); NLY01 (2,5 mg); and placebo	85; 85; and 84	54 (64); 60 (71); 52 (62)	60.6 ± 10.0; 62.1 ± 9.0; and 61.8 ± 8.1	All the time (untreated PD)	22.0 ± 8.2; 22.7 ± 8.1; and 22.3 ± 9.1	82/3; 82/3; and 80/4	6-month follow-up
Meissner et al. 8 (2024)	Early PD	Lixisenatide and placebo	78; 78	44 (56) 48(62)	59.5 ± 8.1; 59.9 ± 8.4	At least 12 hours before evaluation	NR	NR	6-, 12-, and 14-month follow-up

Abbreviations: MDS-UPDRS, Movement Disorder Society Unified Parkinson's Disease Rating Scale; NR, not reported; PD, Parkinson's disease.

### MDS-UPDRS part III

**Figure 2:**
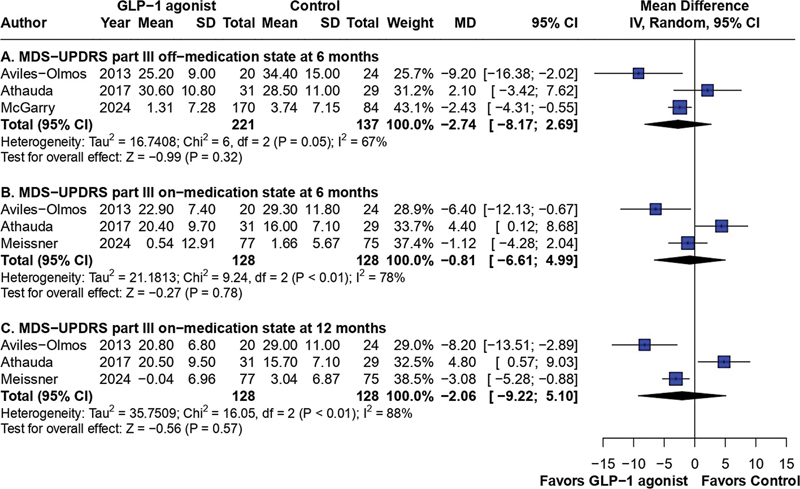
Forest plot of part III of the Movement Disorder Society Unified Parkinson Disease Rating Scale (MDS-UPDRS) in the off-medication state at 6 months ( **A** ); outcomes according to part III of the MDS-UPDRS in the on-medication state at 6 ( **B** ) and at 12 months ( **C** ).

A total of 3 studies investigated the effect of GLP-1 receptor agonists in part III of the MDS-UPDRS in the off-medication state after 6 months, including 358 patients (221 patients in the GLP-1 group and 137 in the control group). The meta-analysis did not reach statistical significance, providing an MD of −2.74 (95%CI: −8.17–2.69; *p* = 0.32; I ^2^ = 67%), as shown in Figure 2 .

For the analysis of part III of the MDS-UPDRS in the on-medication state at 6 months, we found 3 articles that met our eligibility criteria and included 256 patients (128 in the GLP-1 group and 128 in the control group) providing an MD of −0.81 (95%CI: −6.61–4.99; *p* = 0.78; I ^2^ = 78%), which was not a significant result. At 12 months, the same 3 studies, with a total sample size of 256 patients, provided an MD of −2.06 (95%CI: −9.22–5.10; *p* = 0.57; I ^2^ = 88%), still showing no improvement in the motor examination for GLP-1 users, as reported in Figure 2 .

The additional meta-analysis at 14 months of part III of the MDS-UPDRS in the off-medication state also showed no significant improvement, with an MD of −2.41 (95%CI: −7.24–2.43; *p* = 0.33; I ^2^ = 58%), as reported in **Supplementary Material Figure S1** (online only).

Neither were GLP-1 agonists superior to placebo in reducing the motor symptoms of PD in the subgroup analysis in any of the time points evaluated. However, the heterogeneity among the studies remained high ( **Supplementary Material Figure S2** ; online only).

### MDS-UPDRS Part I

**Figure 3:**
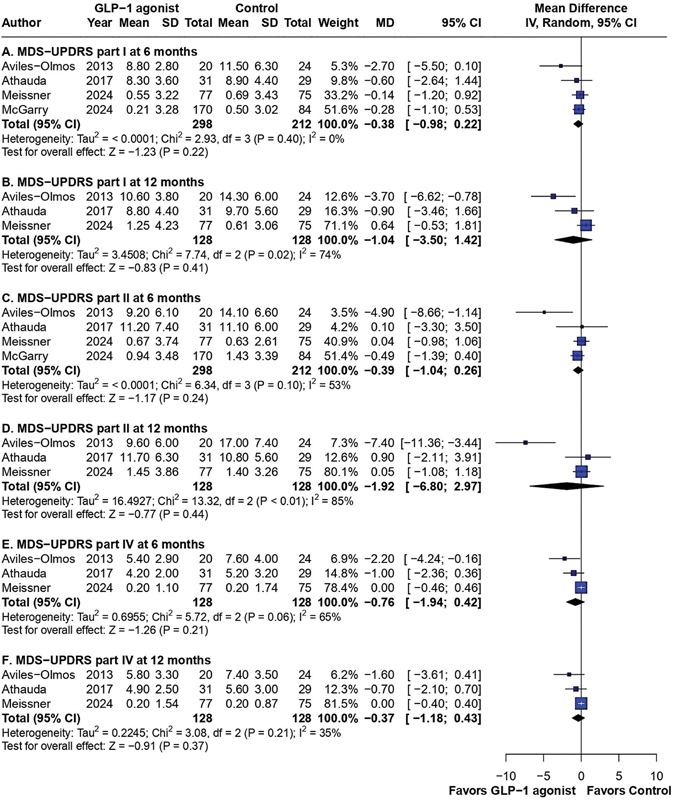
Forest plot of part I of the MDS-UPDRS at 6 ( **A** ) and 12 months ( **B** ); part II of the MDS-UPDRS at 6 ( **C** ) and 12 months ( **D** ); and outcomes according to part IV of the MDS-UPDRS at 6 ( **E** ) and 12 months ( **F** ).

**Figure 4:**
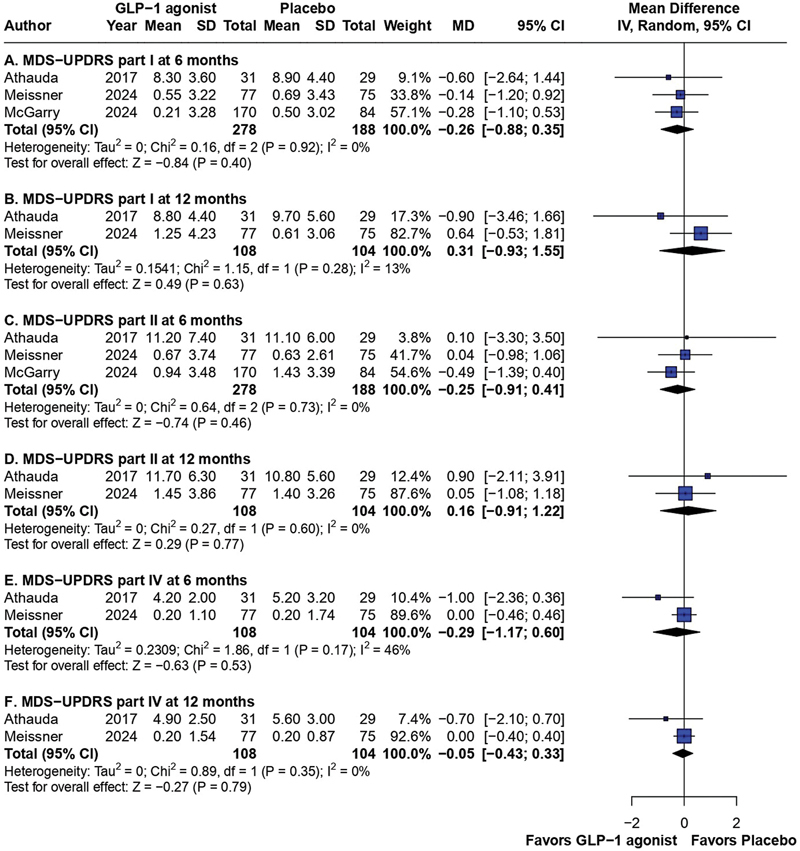
Subgroup analysis of part I of the MDS-UPDRS at 6 ( **A** ) and 12 months ( **B** ); part II of the MDS-UPDRS at 6 ( **C** ) and 12 months ( **D** ); and outcomes according to part IV of the MDS-UPDRS at 6 ( **E** ) and 12 months ( **F** ).

All 4 studies 7 8 16 17 analyzed part I of the MDS-UPDRS at 6 months and included 510 patients. The I ^2^ analysis revealed no significant heterogeneity, but this result was not statistically significant, with an MD of −0.38 (95%CI: −0.98–0.22; *p* = 0.22; I ^2^ = 0%). The 12-month assessment included 3 studies with 256 patients and showed no difference among the groups in non-motor aspects of daily living, with an MD of −1.04 (95%CI: −3.50–1,42; *p* = 0.41; I ^2^ = 74%), as shown in Figure 3 . Although there was high heterogeneity for this endpoint, the subgroup analysis with placebo-controlled trials only showed similar results, with I ^2^ = 13% ( Figure 4 ).

### MDS-UPDRS Part II

All 4 studies 7 8 16 17 assessed changes in part II of the MDS-UPDRS at 6 months, including 510 patients, and no significant differences were observed among the groups, with an MD of −0.39 (95%CI: −1.04–0.26; *p* = 0.24; I ^2^ = 53%). Neither did the analysis at 12 months show any differences in 3 studies ( *n* = 256), with an MD of −1.92 (95%CI: −6.80–2.97; *p* = 0.44; I ^2^ = 85%). The subgroup analysis still showed no difference between placebo and GLP-1 agonists in motor activities of daily living, with no significant heterogeneity (I ^2^ = 0%) for either analysis period ( Figure 4 ).

### MDS-UPDRS Part IV

The pooled analysis showed no significant benefit of GLP-1 agonists in part IV of the MDS-UPDRS at 6 and 12 months. At 6 months, 3 studies ( *n* = 256) provided an MD of −0.76 (95%CI: −1.94–0.42; *p* = 0.21; I ^2^ = 65%). At 12 months, the same studies provided an MD of −0.37 (95%CI: −1.18–0.43; *p* < 0.37; I ^2^ = 35%), as shown in Figure 3 . Neither did the subgroup analysis show improvement in the scores compared with the placebo group, with considerably lower levels of heterogeneity (I ^2^ = 46% at 6 months; I ^2^ = 0% at 12 months), as shown in Figure 4 .

### PDQ-39

All 4 studies 7 8 16 17 evaluated the PDQ-39 at 12 months and reported no statistical difference. This analysis provided an MD of −0.75, and the I ^2^ analysis revealed no significant heterogeneity (95% CI: −3.06–1.55; *p* = 0.52; I ^2^ = 0%), as reported in **Supplementary Material Figure S3** (online only). Neither did placebo-controlled trials show differences in QoL between the placebo and GLP-1 agonist groups ( **Supplementary Material Figure S4** ; online only).

### Adverse effects

The pooled analysis showed that GLP-1 users had a higher risk of nausea, vomiting, constipation, and weight loss when compared with controls. The risk of diarrhea was similar between the groups ( **Supplementary Material Figure S5** ; online only).

### Quality assessment of the included studies

**Supplementary Material Figure S6** (online only) provides a detailed overview of the risk of bias assessment, which was used to evaluate the quality of the studies included in the current meta-analysis. We considered one study 16 to be at a high risk of performance bias because it was not possible to blind participants and staff to treatment groups, as the study authors considered the cost of producing a placebo pen prohibitive. They 16 reported that the participants might have been able to detect their treatment allocation because of adverse events, including injection-site reactions. All other studies 7 8 17 were judged to be at low risk.

## DISCUSSION

The present systematic review and meta-analysis of 4 RCTs 7 8 16 17 involving 514 patients compared GLP-1 agonists with placebo or the standard PD treatment. The key findings include:

- The use of GLP-1 agonists was not associated with an improvement in the motor or non-motor features of PD as assessed by parts I, II, III, and IV of the MDS-UPDRS at 6 and 12 months of follow-up;
- Quality of life, as assessed by the PDQ-39, showed no significant differences between the groups; and
- A higher risk of gastrointestinal side effects (nausea, vomiting, constipation) and weight loss was observed with the use of GLP-1 agonists.

A subgroup analysis specifically comparing GLP-1 agonists and placebo further confirmed the lack of clinical benefit of the intervention in all of these efficacy outcomes and significantly reduced the heterogeneity of the results, enhancing the robustness of the conclusions.

Intensive research into new treatment strategies for PD has highlighted GLP-1 receptor agonists as potential new therapeutic agents. 18 The possibility that these drugs may yield neuroprotective benefits is supported by robust preclinical studies suggesting that they affect pathological pathways relevant to PD. These mechanisms include inhibiting inflammation, 19 20 promoting mitochondrial biogenesis, 21 22 stimulating neurogenesis, restoring dopaminergic neurotransmission 23 and neuronal insulin signaling, 24 and preventing alpha-synuclein aggregation. 25

The MDS-UPDRS is the rating scale most widely used to describe and quantify the signs and symptoms of PD. 26 Part III represents the objective assessment of parkinsonism by a clinician-conducted motor examination. Although the off-medication state measure provides a better insight into disease severity than the on-medication scores, additional variability in scores may occur due to differences in the time since the last dose of PD medication. 17 Therefore, each study's definition of the off-medication state is crucial to enable a pooled analysis. All studies 7 8 16 17 used similar concepts ( Table 1 ), and, in one, 7 the authors performed the evaluation exclusively in the off-medication state due to its early untreated PD population.

Nevertheless, in the current meta-analysis, we found no significant differences among the groups in Part III at 6 and 12 months of follow-up, regardless of medication status. In contrast, the authors of a previous meta-analysis 6 on exenatide found mixed results in Part III, with improvement only in the off-medication state after 12 months, which was not confirmed by the on-medication analysis. Our findings suggest that recently-published trials 7 8 with lixisenatide and NLY01, despite involving larger samples and PD patients in earlier stages, who could potentially benefit from a neuroprotective agent, lacked the statistical power to confirm a benefit in this aspect of the assessment.

Determining whether some or all the described mechanisms of action of GLP-1 receptor agonists alter the natural history of PD, or if they merely produce symptomatic effects upon exposure, is a challenge for clinical trials. 27 Knowing this, the present meta-analysis also sought to investigate whether, after an 8- to 12-week wash-out period, there would be lower levels of impairment in the scores on part III of the MDS-UPDRS. This result did not show a sustained effect of these drugs after discontinuation, in sharp contrast to the motor and cognitive benefits described up to 12 months after cessation of exenatide use in the single-blinded study conducted by Aviles-Olmos et al., 28 which we considered to be at a high risk of bias.

However, it is impossible to dismiss the potential benefits of GLP-1 agonists on the motor symptoms of PD in specific patient profiles. For example, subgroup analyses from 2 trials 7 8 included in the current meta-analysis, comparing participants younger than 60 years of age and those aged 60 years or older, showed lower scores on part III of the MDS-UPDRS in the younger group. Nevertheless, these are post-hoc analyses from which no definitive conclusions can be drawn. The wide age range of the patients included in the present meta-analysis may have contributed to the high heterogeneity in the scores on p-art III, even when GLP-1 agonists were compared with placebo alone.

In the current analysis, non-motor symptoms, such as apathy, depressed and anxious mood, sleep disturbance, or cognitive impairment, all assessed by part I of the MDS-UPDRS, did not show improvement at 6 or 12 months in trials of different GLP-1 receptor agonists including exenatide, NLY01, and lixisenatide. 7 8 16 17 It is possible that different GLP-1 receptor agonists may influence these results, as preliminary reports from a PD trial 29 involving liraglutide intervention appear to contradict the results herein described, despite 12 early withdrawals and the fact that the research has not been peer-reviewed yet.

Motor aspects of experiences of daily living, as measured by part II of the MDS-UPDRS, showed no improvement in participants receiving a GLP-1 agonist at any follow-up time point, nor did the motor complication outcomes (part IV of the MDS-UPDRS). The latter is used to assess dyskinesia and motor fluctuations. Epidemiologic studies 30 have shown that disease progression is an important factor in the development of these motor complications, so the absence of RCTs including patients with Hoehn and Yahr Scale scores ≥ 3 (higher functional disability), may have contributed to the observed lack of effect.

A critical finding of the current meta-analysis is the persistence of non-significant differences when comparing GLP-1 agonists with placebo ( Figure 4 ), accompanied by a substantial reduction in heterogeneity to statistically insignificant levels among the studies in parts I, II, and IV of the MDS-UPDRS. The study by Aviles-Olmos et al., 16 in which the participants were aware of their treatment allocation (an open-label study from the patient's perspective), contributed significantly to the observed heterogeneity in these results ( Figure 3 ), as it was the only study included in the present meta-analysis in which the intervention group had significantly lower scores compared with the control group. Therefore, the positive results of this RCT in parts I, II, and IV of the MDS-UPDRS may have been influenced by the lack of blinding to placebo.

The PDQ-39 is the most widely used tool to assess health-related QoL in PD. 31 Previous studies 32 33 have shown that both motor performance and non-motor symptoms strongly impact QoL. Therefore, reflecting the equivalence among groups in MDS-UPDRS scores, the PDQ-39 analysis also failed to demonstrate better health status using GLP-1 receptor agonists. The adverse effect profile was as expected for this class of agent, with a higher risk of gastrointestinal symptoms (nausea, vomiting, and constipation) compared with the control group. Only one serious adverse event (pancreatitis) possibly related to GLP-1 agonists, specifically lixisenatide, was observed in one of these studies. 8 The adverse effects found may limit the acceptance and adherence to treatment, compromising the clinical usefulness of these agents in the daily practice.

The present study has major limitations. First, the results may not apply to all PD patients, as the stages of PD ranged from early to moderate. Another limitation is that the analysis did not include enough studies to draw a confident conclusion, and 2 studies 16 17 had small sample sizes and some baseline imbalances between the groups, highlighting the need for further trials with larger sample sizes. Finally, there was significant heterogeneity in the scores on parts I, II, III, and IV of the MDS-UPDRS. To address this, a subgroup analysis comparing the GLP-1 agonist group to a placebo control group was performed, which resulted in reduced heterogeneity in parts I, II, and IV. However, the forest plot of part III still showed substantial heterogeneity. This may be due to the variability in the initial stage of the disease across the studies, different concomitant medications for PD, and the wide range of patients' ages. Thus, gathering such a heterogeneous group of patients and expecting a uniform response to GLP-1 receptor agonists may seem somewhat counterintuitive. Finally, the total number of patients included those receiving different GLP-1 analogues at various doses, which contributes to the heterogeneity found and limits the interpretation of the data regarding the class effect of these drugs.

The current is meta-analysis of 514 PD patients showed that the use of GLP-1 agonists was not associated with an improvement in PD motor or non-motor features, as assessed by parts I, II, III, and IV of the MDS-UPDRS. This finding suggests that, despite the theoretical neuroprotective properties of GLP-1 agonists, these agents did not demonstrate a measurable clinical impact on PD manifestations within the studied period. The safety profile was as expected, with a higher risk of gastrointestinal symptoms. Future double-blinded RCTs should include longer duration of treatment to establish longer-term effects of the intervention, since we were limited to a one-year period in the present analysis, and PD has a progressive nature. Younger patients at an even significantly earlier stage of PD should be included in future trials, as they might be more likely to show benefits from neuroprotective agents. Additional high-quality studies are needed to draw more robust conclusions about this treatment.
